# Clinically Meaningful Tumor Reduction Rates Vary by Prechemotherapy MRI Phenotype and Tumor Subtype in the I-SPY 1 TRIAL (CALGB 150007/150012; ACRIN 6657)

**DOI:** 10.1245/s10434-013-3038-y

**Published:** 2013-06-19

**Authors:** Rita A. Mukhtar, Christina Yau, Mark Rosen, Vickram J. Tandon, The I-SPY 1 TRIAL and ACRIN 6657 Investigators, Nola Hylton, Laura J. Esserman

**Affiliations:** 1 University of California, San Francisco, San Francisco, CA USA; 2 University of Pennsylvania, Philadelphia, PA USA

## Abstract

**Purpose:**

This study was designed to determine (1) rates of clinically meaningful tumor reduction in breast tumor size following neoadjuvant chemotherapy (NAC), (2) which receptor subtypes and MRI phenotypes are associated with clinically meaningful tumor reduction, and (3) whether MRI phenotype impacts concordance between pathologic and MRI size.

**Methods:**

We analyzed data from the I-SPY TRIAL, a multicenter, prospective NAC trial. Reduction in tumor size from >4 to ≤4 cm was considered clinically meaningful, as crossing this threshold was considered a reasonable cutoff for potential breast conservation therapy (BCT). MRI phenotypes were scored between one (well-defined) and five (diffuse) on pre-NAC MRIs.

**Results:**

Of 174 patients with tumors >4 cm, 141 (81 %) had clinically meaningful tumor reduction. Response to therapy varied by MRI phenotype (*p* = 0.003), with well-defined phenotypes more likely than diffuse phenotypes to have clinically meaningful tumor shrinkage (91 vs. 72 %, *p* = 0.037). Her2+ and triple-negative (Tneg) tumors had the highest rate of clinically meaningful tumor reduction (*p* = 0.005). The concordance between tumor diameter on MRI and surgical pathology was highest for Her2+ and Tneg tumors, especially among tumors with solid imaging phenotypes (*p* = 0.004).

**Discussion:**

NAC allows most patients with large breast tumors to have clinically meaningful tumor reduction, meaning response that would impact ability to undergo BCT. However, response varies by imaging and tumor subtypes. Concordance between tumor size on MRI and surgical pathology was higher in well-defined tumors, especially those with a Tneg subtype, and lower in HR+ diffuse tumors.

**Electronic supplementary material:**

The online version of this article (doi:10.1245/s10434-013-3038-y) contains supplementary material, which is available to authorized users.

Neoadjuvant chemotherapy (NAC) is used increasingly for breast cancer treatment, with two main benefits: it offers the ability to monitor response to treatment, where pathologic complete response (pCR) is prognostic, and it can result in downstaging of tumor and breast conservation treatment (BCT) or eliminate the need for postmastectomy radiation in the setting of pCR.^1^–^6^ Many factors influence the choice of surgical procedure after NAC: patient preference, tumor appearance, hormone receptor (HR) and Her2 expression status, and treatment response.^7^,^8^ Whereas the post-NAC MRI often is used to determine whether BCT is possible, investigators note that the pre-NAC MRI influences surgeons’ recommendations, regardless of tumor appearance after NAC.^9^ Because clinicians and patients seek to avoid reexcision, it is important to understand the reliability of the postchemotherapy MRI.^10^–^13^

Physical examination, ultrasound, and mammography have only moderate accuracy in predicting residual disease, whereas MRI longest diameter and volumetric measurements are the most accurate measures after chemotherapy.^14^–^17^ Despite this, both false positives and negatives remain. A better understanding of imaging reliability and which features predict successful BCT could affect surgical management.^18^,^19^

We previously identified that MRI phenotypes (solid and well-contained vs. diffuse and infiltrative) correspond with degree of response to NAC and predict the ability to achieve breast conservation.^20^ In this study, we investigated whether MRI phenotype and receptor subtype predicted rates of clinically meaningful tumor reduction in a larger cohort with imaging and molecular data. Surgeon comfort with attempting BCT varies and was not mandated; our primary endpoint therefore was crossing the threshold from >4 cm to tumor size of ≤4 cm, a reasonable cutoff for potential BCT. We also investigated whether the correlation between tumor size on post-NAC MRI and surgical pathology differed by MRI phenotype and receptor subtype.

## Methods

### Patients

Patients received anthracycline-based chemotherapy, followed by a taxane regimen on the I-SPY 1 TRIAL (CALGB 150007/150012, ACRIN 6657).^1^,^2^ Herceptin was used neoadjuvantly in Her2+ patients after 2005. This study was approved by the UCSF institutional review board.

### MRI Technique

**Fig. 1:**
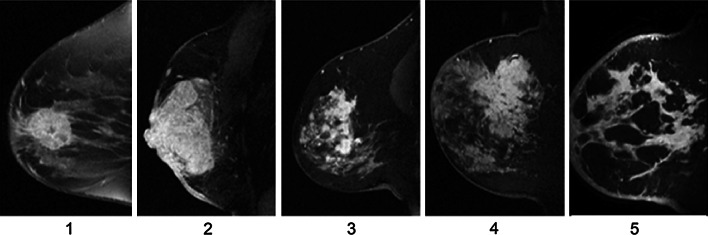
Examples of each of the five MRI phenotypes: **1** well defined, unicentric mass; **2** well defined, multilobulated mass; **3** area enhancement with nodularity; **4** area enhancement without nodularity; **5** septal spreading

Contrast-enhanced MRI was performed on 1.5T MRI scanners using dedicated breast radiofrequency coils (details previously described).^21^ Unilateral images were acquired using 3D, fat-suppressed, T1-weighted spoiled gradient echo (SPGR) imaging with spatial resolution ≤1 mm/pixel in-plane and ≤2.5-mm slice thickness. Pre- and postgadolinium (0.1 mmol/kg) imaging was performed at prespecified temporal resolution to achieve imaging at ~2.5 and 7.5 min after contrast administration. Tumors were assigned one of five MRI phenotypes based on pre-NAC imaging: 1—well defined, unicentric mass; 2—well defined, multilobulated mass; 3—area enhancement with nodularity; 4—area enhancement without nodularity; 5—septal spreading (Fig. 1). Pre-NAC MRI phenotype was determined by a centrally trained breast radiologist at each study site.

### Determination of Tumor Marker Subtypes

Core biopsies were obtained at each site before NAC, and immunohistochemical and genomic markers were performed as previously described.^1^,^2^

### Postsurgical Pathology Analysis

Seven study pathologists were trained on evaluation of gross and microscopic sections using a standardized tool: the residual cancer burden method.^22^ Training was done by Dr. Fraser Symmans, who reviewed the first five cases from each pathologist. An electronic tool was built to capture extent of disease. Pathology size was re-reviewed and longest diameter from this central re-review was used as the largest size.

### Definition of Clinically Meaningful Tumor Reduction

Subjects were considered potentially eligible for BCT before receiving NAC if the tumor was ≤4 cm on both pre-NAC clinical examination and pre-NAC MRI. This cut point was chosen based on its use in the National Surgical Adjuvant Breast and Bowel Project B-06.^23^ Patients with tumors >4 cm in size before NAC were considered to have a clinically meaningful tumor reduction if the tumor was ≤4 cm on surgical pathology. Discrepancy between post-NAC MRI and surgical pathology was defined as a difference in longest tumor diameter of ≥2 cm (cutoff based on internal consensus). Employment of stricter cutoffs (up to 1.5 cm) did not significantly alter our findings.

### Statistical Analysis

As most variables were not normally distributed, median values and ranges are reported. The Kruskal–Wallis test was used to compare continuous variables, and contingency tables with the χ^2^ test were used for categorical variables.^24^ MRI phenotypes were analyzed as five distinct categories, as well by dichotomization into well-defined versus diffuse categories. *p* values were two-sided, and *p* < 0.05 based on contingency table χ^2^ statistical test was considered significant.^25^

## Results

### Clinical Characteristics

Of the 221 subjects in the I-SPY TRIAL, 198 had data available for pre- and posttreatment MRIs, pretreatment clinical examinations, type of surgery, and surgical pathology; 193 had pre-NAC HR and Her2 status available.^1^,^2^ Of the 198 subjects in this study, 24 had tumors ≤4 cm on both pre-NAC clinical examination and pre-NAC MRI.

### Clinically Meaningful Tumor Reduction

Of the 174 subjects with tumors >4 cm on pre-NAC clinical examination and MRI, 141 (81 %) had a clinically meaningful tumor reduction after NAC based on a tumor size ≤4 cm on surgical pathology. Sixty-one of these 174 (35 %) subjects received BCT. Of the 141 subjects whose tumors shrank to ≤4 cm, 52 (37 %) received BCT, 2 (1.4 %) had attempted BCT but subsequently required mastectomy for positive margins, and 87 (62 %) underwent mastectomy. The most common reasons for not receiving BCT included multicentric disease (22 %) and patient choice (22 %; Supplementary Table 1).

**Fig. 2:**
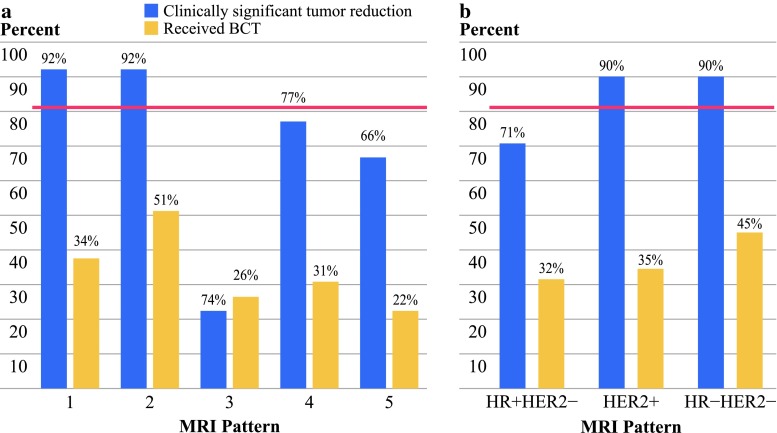
**a** Bar plot showing percentage of patients who had clinically meaningful tumor reduction (*blue*) and who received BCT (*yellow*) after NAC by MRI phenotype; pink line represents the average rate of clinically meaningful tumor reduction (81 %). Subjects with well-defined MRI phenotypes (*1* and *2*) were more likely to have a clinically meaningful tumor reduction and receive BCT after NAC. **b** Bar plot showing same data by receptor subtype. Her2+ and Tneg tumors had a higher likelihood of having clinically meaningful tumor reduction after NAC compared to HR+/Her2− tumors; however, the actual rates of receiving BCT do not differ by subtype

The response to NAC varied by MRI phenotype (*p* = 0.037). Patients with well-defined pre-NAC MRI phenotypes (Fig. 1) had higher rates of clinically meaningful tumor reduction than those with diffuse phenotypes (92 vs. 72 %; Fig. 2a). The rates of BCT were higher in the well-defined phenotypes compared with the diffuse phenotypes (47 vs. 27 %, *p* = 0.023; Fig. 2a).

The rate of clinically meaningful tumor reduction also varied by receptor subtype (*p* = 0.005). Her2+ and Tneg (HR−/Her2−) tumors had higher rates compared with the HR+/Her2− group (Fig. 2b). However, there was no significant difference in BCT rates (Fig. 2b). Analysis using a 3-cm cutoff for clinically meaningful tumor reduction yielded similar results.

### MRI/Pathology Concordance

**Fig. 3:**
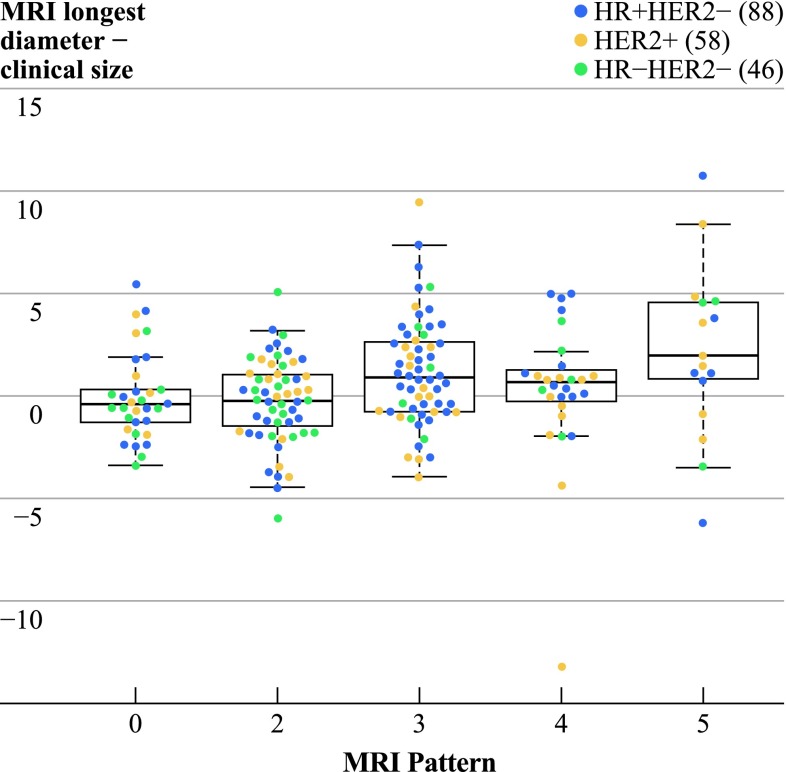
*Vertical axis* shows the difference in centimeters between tumor diameter on pre-NAC MRI and tumor diameter palpated on pre-NAC physical examination. *Horizontal axis* shows results by MRI phenotype. Overall, palpation underestimated tumor size in the diffuse tumors and slightly overestimated in solid tumors. Note that one Tneg case was left out of this analysis

Pretreatment tumor size varied by MRI phenotype pattern (*p* = 0.002; Supplementary Table 2). In addition, tumor diameter on pre-NAC MRI differed significantly from tumor diameter by palpation (paired Wilcoxon rank-sum test, *p* = 0.01) and this difference varied by imaging phenotype (*p* = 0.003) with a median size difference of −0.4, −0.2, 0.9, 0.65, and 2 cm for phenotypes 1–5, respectively. Clinical diameter tended to be larger than MRI diameter in solid tumors. In diffuse phenotypes, the MRI size was larger than by clinical exam (Fig. 3). One Tneg tumor was excluded from this analysis because the tumor took up the entire breast, so preoperative size was not recorded.

**Fig. 4:**
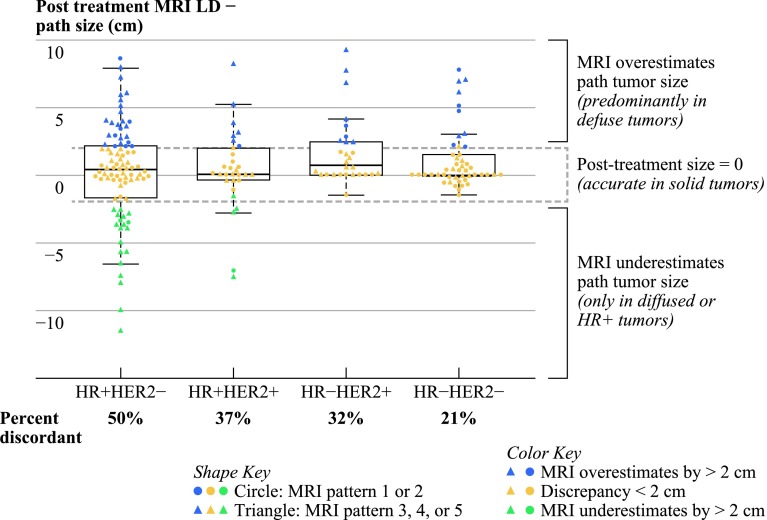
*Vertical axis* shows the difference in centimeters between post-NAC MRI longest diameter and tumor size on surgical pathology. Solid tumors had smaller size discrepancies than diffuse tumors. MRI underestimated path tumor size only in diffuse or HR+ tumors. For cases where MRI was accurate (<2 cm difference), there were 72 solid and 51 diffuse cases. For cases were MRI overestimated path tumor size, there were 16 solid and 36 diffuse cases. MRI most often overestimated the size of diffuse tumors, except in the case of HR+/Her2− tumors. *Circles* represent MRI phenotypes *1* and *2*; *Triangles* are phenotypes *3*, *4*, and *5*

The post-NAC MRI longest diameter measurements differed from diameter measured on surgical pathology (Fig. 4). Of the 198 subjects analyzed, 75 (38 %) had a discrepancy of ≥2 cm between size on imaging and pathology. Of the 75 discrepant cases, the tumor size on post-NAC MRI was greater than pathology in 52 cases (69 %) and smaller in 23 cases (31 %). There were 18 cases (9 %) in which pathology showed no tumor, but post-NAC MRI tumor size was ≥2 cm, and 7 cases (3.5 %) in which post-NAC MRI showed no tumor, but pathology showed ≥2 cm of tumor. MRI/pathology concordance varied by tumor subtype (*p* = 0.004), with size discrepancies present in half of all HR+/Her2− tumors but lower discrepancy rates in Her2+ and Tneg tumors. Underestimation of disease by >2 cm was rare (4.3 %) in solid MRI tumor patterns. In cases where the post-NAC MRI underestimated the tumor size, all tumors were either diffuse and/or HR+/Her2− or Her2+, and none were Tneg. Within each marker subtype, diffuse tumors were more likely to have size discrepancies. Overall, diffuse HR+ Her2− tumors were most likely to have discrepancies between post-NAC MRI and surgical pathology (Fig. 4). Similar trends were observed among patients who received BCT (80/198 total patients analyzed), with the highest discrepancy rates in HR+ HER2− diffuse cases (31 vs. 19 % all others) and MRI underestimation of tumor size occurring only in diffuse and/or HR+ cases. Additionally, employing stricter cutoffs for discrepancy (up to 1.5 cm) did not alter these findings.

### MRI Phenotype and Tumor Subtype

**Fig. 5:**
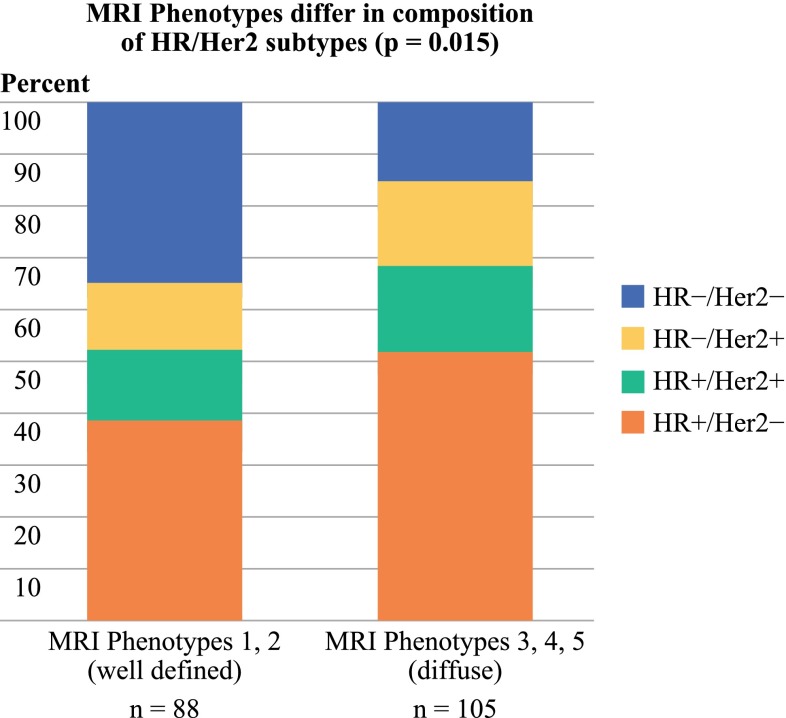
Whereas the relative proportions of tumor subtypes varied between the well-defined and diffuse MRI phenotypes, both groups contained all four receptor based subtypes

The most common patterns were MRI phenotypes 2 and 3 (Table 1). There were significant differences in the receptor subtype distribution for well-defined versus diffuse MRI categories (*p* = 0.015 by χ^2^ test). The Tneg (HR−/Her2−) group made up a larger proportion of the well-defined MRI phenotypes (35 %) than the diffuse phenotypes (15 %). The diffuse patterns had a higher proportion of HR+ cases (68 %) than the well-defined patterns (52 %; Fig. 5). However, all phenotypes were represented in each receptor subtype.

**Table 1:** Patient and tumor characteristics: age, stage, histology, grade, chemotherapeutic regimens, HR/Her2 marker status, surgical treatment received, and MRI phenotype

	Total (*n* = 198)
	Number (%)
Median age (range)	48.5 (26.7–68.8)
Stage at presentation	
I	3 (1.5)
II	93 (47)
III	88 (44.4)
Inflammatory	14 (7)
Histology	
Necrosis	3 (1.5)
Ductal	158 (80)
Lobular	18 (9)
Mixed ductal-lobular	7 (3.5)
Other/not available	12 (6)
Grade	
1	14 (7)
2	90 (45.5)
3	90 (45.5)
Indeterminate	4 (2)
Chemotherapy regimen	
Doxorubicin (A)/cyclophosphamide (C)	9 (4.6)
Doxorubicin (A)/cyclophosphamide (C)/taxane	171 (86.4)
Doxorubicin (A)/cyclophosphamide (C)/taxane/herceptin	16 (8)
Doxorubicin (A)/cyclophosphamide (C)/taxane/other	2 (1)
Marker subtype	
HR+/Her2−	88 (44.4)
Her2+	58 (29.7)
HR−/Her2−	47 (23.7)
Unavailable	5 (2.5)
Surgical treatment	
Lumpectomy	80 (40.4)
Lumpectomy followed by mastectomy	2 (1)
Mastectomy	116 (59)
MRI Phenotype	
1 well defined unicentric mass	33 (16.7 %)
2 well defined multilobulated mass	59 (29.8 %)
3 area enhancement with nodularity	60 (30.3 %)
4 area enhancement without nodularity	28 (14.1 %)
5 septal spreading	18 (9.1 %)
Potentially Eligible for BCT	
Pre-NAC (palpation and MRI <4 cm)	
Yes	24 (14 %)
No	150 (86 %)
Post-NAC (pathology size <4 cm)	
Yes	141 (81 %)
No	33 (19 %)

## Discussion

Overall, the results in this prospective cohort of patients confirm our previous findings that tumor morphology, captured by MRI phenotype, and tumor subtype affect rates of achieving clinically meaningful tumor reduction after NAC.^26^ Patients with well-defined MRI phenotypes and those with Her2+ and Tneg tumors were more likely to have tumor shrinkage to ≤4 cm. We and others have found an improved correlation between post-NAC MRI and surgical pathology in tumors that are well-defined by imaging.^27^ Together, these findings suggest that MRI phenotype may be used in conjunction with tumor subtype to set appropriate expectations before undergoing NAC.

MRI phenotype and tumor subtype likely reflect biological differences between tumors. Other phenotypic features are associated with different tumor subtypes. Basal-like breast tumors have distinct histologic and immunotypic properties, with characteristics, such as central scar, tumor necrosis, spindle cells, squamous metaplasia, high mitotic count, and high nuclear to cytoplasmic ratio.^28^,^29^ Most of these Tneg tumors have mass-like imaging patterns on MRI.^30^,^31^ Even on mammogram and ultrasound, breast cancer subtypes have imaging characteristics, with Tneg cancers more likely to have smooth margins.^32^,^33^

MRI phenotype and marker status influenced the likelihood of size discrepancies between imaging and pathology. Similarly, Chen et al.^9^,^34^ found that MRI was less accurate in tumors that present as non-mass enhancement on MRI, and in another study suggest that MRI can be used more successfully to plan BCT in Her2+ patients. Others also have reported that post-NAC MRI appears to be less accurate in ER+ tumors and most accurate in Tneg or Her2+ tumors and that pre-NAC tumor size and response also impact accuracy.^34^–^37^ Although some have reported the highest accuracy of MRI for Her2− disease, not knowing HR status and differences in rates of traztuzumab use could potentially account for the disparate results.^38^ Benign proliferative processes can enhance on MRI and are difficult to differentiate from low-grade, ER+ DCIS lesions. False-positive MRI enhancement may reflect a spectrum of change within high-risk tissue, possibly explaining why it is difficult to distinguish residual tumor size in ER-positive patients, especially with diffuse disease.^39^,^40^

We previously reported differences in response to NAC based on the five MRI phenotypes described. Patients with well-circumscribed masses had the greatest response to NAC.^20^ In the current study, the majority of patients (81 %) achieved shrinkage to tumor size ≤4 cm, whereas we previously found only 47 % achieved enough shrinkage to be potentially eligible for BCT.^26^ This was likely due to the addition of taxane, which doubles the pCR rate compared with doxorubicin alone.^41^ For tumor subtypes, adjusting for pre-NAC tumor size did not change our results. For MRI phenotype, we found that size and phenotype were associated, because diffuse tumors will necessarily occupy a larger space. The larger size of these diffuse tumors could influence the ability to reach the threshold of ≤4 cm, but separating the contribution of phenotype from size is not possible in this study. The tumor response to NAC also can affect MRI accuracy, with good correlation between MRI and pathology noted in tumors with extreme responses (either complete or none), and worse correlation among partial responders.^42^,^43^

For the MRI phenotypes, the well-defined groups had higher BCT rates, but no difference was seen among tumor subtypes. Whereas patient choice was a factor in 22 % of cases that were potentially candidates for but did not receive BCT, other factors, such as physician recommendations, could play a role in surgical decisions. Knowing the accuracy of MRI could alter these recommendations. Interestingly, post-NAC MRI longest diameter showed a stronger association with surgical procedure than tumor size on surgical pathology or post-NAC mammographic longest diameter among the 175 patients assessed by all three methods (Wilcoxon rank-sum test, *p* = 0.001, 0.17, and 0.02 respectively). There was no significant difference in local recurrence or recurrence free survival between subjects who received BCT and those who received mastectomy.^44^

More than one third of patients had a size discrepancy ≥2 cm between the post-NAC MRI and surgical pathology. Some have suggested that overestimation on MRI could be a result of taxane causing increased vascular permeability and gadolinium uptake, or related to an inflammatory infiltrate or necrosis.^43^,^45^ We found more discrepancies in the diffuse tumor phenotypes, which likely reflects increased difficulty in measuring tumor diameter. These discrepancies were particularly notable in the diffuse HR+/Her2− tumors and make it more difficult to set expectations based on post-NAC MRI in these tumor types. However, in the setting of HR− tumors of solid phenotypes, post-NAC imaging did not underestimate residual tumor size. When MRI showed a pCR, the surgical pathology was concordant.

The strengths of this study include central assessment of HR/Her2 status and the consistent timing of MRIs. There were dedicated breast radiologists at each site who underwent centralized training to validate the MR phenotypes. Size assessment can be somewhat subjective, however, especially for diffuse tumors. Adding tumor volume measurements may help to decrease the chance of overestimating the tumor size compared to surgical pathology. Despite these limitations, however, the findings are consistent with those reported in the literature, with the additional finding that MRI size estimates are less likely to correspond well with pathology for HR+ diffuse tumors.

These findings have clinical implications. Whereas the majority did not attain a pCR, most patients attained clinically meaningful tumor reduction. The MRI phenotype and tumor subtype can inform the discussion about the likelihood of achieving enough response to be potentially eligible for BCT. Whether this information would increase rates of receiving BCT is unknown, but increased understanding, particularly of the accuracy of post-NAC MRI, could impact recommendations and patient decisions.

Overall, many more patients have clinically meaningful tumor reductions than have a pCR. Although the reasons for not receiving BCT are complex, there is likely room for improvement in offering BCT to more patients. We are currently developing an algorithm based on biologic and MRI features to help determine the chances of having a clinically meaningful tumor reduction and the likely accuracy of MR post-NAC to guide this decision-making process.

## Electronic supplementary material

Below is the link to the electronic supplementary material.

Supplementary material 1 (DOC 99 kb)
