# Supplementary material for: Clinically Meaningful Tumor Reduction Rates Vary by Prechemotherapy MRI Phenotype and Tumor Subtype in the I-SPY 1 TRIAL (CALGB 150007/150012; ACRIN 6657)
Source: Ann Surg Oncol. 2013 Jun 19;20(12):3823–30. doi: 10.1245/s10434-013-3038-y (PMC3824937; doi:10.1245/s10434-013-3038-y)
Supplement: Supplementary file 1 — Supplementary material 1 (DOC 99 kb) [file 10434_2013_3038_MOESM1_ESM.doc]

**Supplementary Table 1 Reasons for lack of BCT in patients with tumors ≤ 4 cm on surgical pathology (n = 87).** Although 141 subjects had tumors ≤ 4 cm in size after receiving NAC, making them potentially eligible for BCT, 87 of these patients still underwent mastectomy. The most common reasons for lack of BCT in these subjects were patient choice and the presence of multicentric disease on pre-NAC MRI.

|  | **Subjects (%)** |
| --- | --- |
| **Patient choice** | 19 (22%) |
| **Multicentric disease** | 19 (22%) |
| **Inflammatory disease** | 13 (15%) |
| **Institutional norm** | 12 (13.8%) |
| **Specific anatomy** | 11 (12.6%) |
| **Diffuse microcalcifications** | 4 (4.6%) |
| **Other** | 9 (10.3%) |

**Supplementary Table 2 Clinical and pathological features by MRI phenotype**

The MRI phenotypes varied by tumor size both on pre-NAC imaging, and post-NAC surgical pathology.

|  | 1 | 2 | 3 | 4 | 5 | p-value (Kruskal Wallis, unless indicated) |
| --- | --- | --- | --- | --- | --- | --- |
| N | 33 | 59 | 60 | 28 | 18 |  |
| Age (mean, years) | 50.4 | 47 | 48.8 | 47.3 | 46 | 0.3 (ANOVA) |
| Pre-chemo clinical tumor size (median, range, cm) | 5, 0-12.5 | 5, 2-13 | 6.25, 1.5-14 | 6.75, 3-25 | 8, 3-18 | 0.0017 |
| Pre-chemo MRI longest diameter (median, range, cm) | 4.2, 2.2-11.5 | 5.6, 2-9 | 7.8, 1.9-16.5 | 8.6, 2-13.8 | 9.8, 6.5-18.4 | 0.0001 |
| Change in MRI longest diameter (median, cm) | -2.5 | -4.1 | -4.8 | -4.9 | -6.4 | 0.74 |
| Post-chemo MRI longest diameter (median, range, cm) | 1.7, 0-8.2 | 1.5, 0-8.6 | 3, 0-12.9 | 3.7, 0-11.4 | 3.4, 0-12.6 | 0.02 |
| Tumor size on surgical path (median, range, cm) | 1.4, 0-8.3 | 1, 0-7 | 1.5, 0-15 | 2, 0-13 | 1.95, 0-10 | 0.3 |
| Tumor size on surgical path (median, range, cm) | 1.1, 0-8.3 | | 1.5, 0-14 | | | 0.045 |
